# Home-Based Digital Healthcare Interventions for Dementia: A Systematic Review of Patient and Family Caregiver Outcomes

**DOI:** 10.3390/healthcare14070854

**Published:** 2026-03-27

**Authors:** Mohammed Nasser Albarqi

**Affiliations:** Family and Community Medicine Department, College of Medicine, King Faisal University, Al Hofuf 31982, Saudi Arabia; aalbarqi@kfu.edu.sa

**Keywords:** dementia, family caregivers, digital health, telehealth, home-based care, psychoeducation, cognitive training, telecare, caregiver burden, sleep

## Abstract

**Background:** Home-based digital healthcare interventions are increasingly used to support people living with dementia (PLWD) and their family caregivers. However, evidence regarding their effectiveness across patient and caregiver outcomes remains heterogeneous. **Methods:** This systematic review followed PRISMA 2020 guidelines and was prospectively registered in PROSPERO (CRD420261302166). Six databases (PubMed, Embase, CINAHL, PsycINFO, Web of Science, and Scopus) were searched from January 2000 to October 2025. Randomized and quasi-experimental quantitative studies evaluating home-based or remotely delivered digital interventions for PLWD and/or informal caregivers were included. Risk of bias was assessed using RoB 2 and ROBINS-I. Due to heterogeneity, findings were synthesized narratively. **Results:** Fourteen studies met the inclusion criteria. Interventions included web-based psychoeducation, telecoaching, digital cognitive training, assistive technologies, and multicomponent programs. Caregiver outcomes demonstrated the most consistent benefits, including reduced burden and stress, improved self-efficacy, and improved sleep efficiency in technology-supported trials. For PLWD, small-to-moderate improvements were observed in global cognition and selected neuropsychiatric symptoms, particularly in interactive and personalized programs. Multicomponent interventions combining caregiver education with patient activation and professional feedback showed more durable effects. **Conclusions:** Home-based digital interventions appear feasible and beneficial, particularly for caregiver outcomes. Future large-scale trials with longer follow-up and standardized outcome measures are needed to confirm durability, equity, and cost-effectiveness.

## 1. Introduction

Dementia is a progressive neurocognitive syndrome marked by declines in memory, executive function, language, and social cognition that interfere with independence in everyday activities [1]. While biomedical advances have refined diagnostic criteria and expanded symptomatic treatments, dementia remains a leading cause of disability and dependency in older adults, with substantial personal, social, and economic consequences for patients, families, and health systems [2]. As the condition evolves, neuropsychiatric symptoms—such as agitation, apathy, sleep–wake disruption, and wandering—often emerge alongside functional decline, amplifying risks of caregiver strain, crisis presentations, and premature institutionalization [3]. Because most people with dementia live at home for the majority of their illness trajectory, the household has become the de facto site of long-term care, where family members (typically spouses and adult children) shoulder complex, evolving care tasks with limited formal support [4,5].

Dementia affects an estimated 55 million people worldwide, with nearly 10 million new cases each year, making it one of the fastest growing major causes of disability and dependency among older adults globally. The global prevalence of dementia is projected to rise to 78 million by 2030 and 139 million by 2050, largely driven by population aging. Dementia accounts for significant healthcare and societal burden, with estimated worldwide costs exceeding USD 1.3 trillion annually, including direct medical care, social care, and informal caregiving costs [6]. The impact is felt inequitably across regions, with low- and middle-income countries experiencing the fastest increases in prevalence and carrying a disproportionate share of the caregiving burden.

Family caregiving is associated with elevated stress, role overload, sleep disturbance, depressive symptoms, and reduced quality of life, particularly when responsive behaviors, nighttime awakenings, and safety concerns intensify [7]. These burdens are unevenly distributed: lower socioeconomic status, limited health literacy, rurality, and constrained service access can compound risk, while cultural expectations shape who provides care and how they cope [8]. Health systems, meanwhile, face rising demand for community-based dementia care, heightening interest in scalable, cost-conscious strategies that sustain function, mitigate behavioral and psychological symptoms of dementia (BPSD), and support caregiver well-being [9].

Digital health has emerged as a promising lever to extend dementia care into the home. Broadly defined, home-based digital interventions encompass mobile applications, web portals, telehealth and telecoaching platforms, remote monitoring and telemonitoring, wearable devices, ambient sensors and smart-home technologies, electronic medication supports, conversational agents, and social or assistive robotics used within domestic settings [10,11,12]. These tools can provide education, self-management supports, dyadic coaching, symptom tracking with automated prompts, risk detection (e.g., falls, elopement), and just-in-time decision guidance for caregivers [13]. When paired with clinician oversight—often nurse-led—they may enable proactive adjustments to care plans, earlier responses to deterioration, and targeted behavioral strategies that reduce BPSD triggers. For caregivers, structured psychoeducation, problem-solving modules, peer support forums, and sleep hygiene programs can be delivered asynchronously or synchronously, increasing reach while accommodating the unpredictable rhythms of caregiving [14].

Similar implementation patterns have been observed in epilepsy digital self-management interventions, where telemonitoring and mobile health tools improved adherence and patient engagement but demonstrated variable effects on seizure control and quality-of-life outcomes depending on intervention intensity and integration with clinical care [15].

Mechanistically, digital interventions may influence outcomes through several pathways. First, information and skills acquisition can enhance caregiver self-efficacy, resulting in more consistent application of non-pharmacological strategies that reduce BPSD and improve daily functioning [16]. Second, monitoring and feedback loops (via wearables or ambient sensors) create visibility into activity, sleep, and safety, enabling preventive action rather than crisis response [17]. Third, care coordination and communication platforms can streamline contact with clinicians and services, lowering barriers to timely support [18]. Fourth, behavioral activation and routine scaffolding—for example, reminders for hydration, medication, and exercise—may stabilize daily rhythms and indirectly benefit nocturnal sleep patterns for both patient and caregiver [19]. Finally, emotional and social support delivered digitally (e.g., moderated groups, messaging) can mitigate isolation and perceived burden [20].

Notwithstanding this promise, evidence is heterogeneous. Interventions vary widely in intensity, human support, and theoretical grounding; outcome measures and timepoints differ; and trials often enroll small, selective samples, limiting generalizability and precision [21]. Studies may prioritize patient outcomes while treating caregiver effects as secondary or exploratory, resulting in underpowered analyses for stress, burden, and sleep. Engagement and adherence—critical determinants of real-world effectiveness—are inconsistently measured or reported, and the relationship between dose and outcome is not well characterized [22]. Moreover, implementation questions loom: usability for dyads with sensory or cognitive limitations; equity of access across digital divides; data privacy and ethical oversight in continuous monitoring; and integration with primary care, memory clinics, and community services [23]. The COVID-19 pandemic accelerated telehealth adoption, but sustained use requires evidence on safety, acceptability, and outcomes beyond crisis conditions [24].

Conceptual frameworks from behavior change, self-efficacy theory, person-centered care, and social cognitive theory can guide intervention design and evaluation, yet explicit theoretical alignment is often absent or superficial [25]. Similarly, outcome selection does not always reflect what matters most to dyads—preserving meaningful routines, relational quality, and nighttime respite for caregivers—nor does it consistently employ validated measures sensitive to change in home contexts [26]. As health systems consider scaling digital dementia services, decision-makers need comparative evidence that links specific tool classes and delivery models (e.g., automated vs. nurse-supported) to prioritized outcomes and subgroups [27].

### Aim of the Study

To systematically synthesize and critically appraise evidence on home-based digital interventions for people living with dementia, quantifying their effects on patient outcomes (e.g., BPSD, function, quality of life, sleep, healthcare utilization) and on family caregiver well-being—specifically stress, burden, and sleep—and to identify intervention and context features associated with benefit.

## 2. Materials and Methods

### 2.1. Search Strategy and Selection Criteria

This systematic review was conducted in accordance with the Preferred Reporting Items for Systematic Reviews and Meta-Analyses (PRISMA) 2020 guidelines to ensure methodological transparency, rigor, and reproducibility. The protocol for this review was prospectively registered with the International Prospective Register of Systematic Reviews (PROSPERO; Registration No. CRD420261302166). The protocol specified the research question, eligibility criteria, search strategy, and planned methods of synthesis prior to study selection, ensuring transparency and reducing risk of reporting bias.

The review aimed to synthesize global evidence on the effects of home-based digital interventions on patient outcomes and caregiver well-being in the context of dementia care. A comprehensive literature search was conducted across six major electronic databases: PubMed (MEDLINE), Embase (Elsevier), CINAHL (EBSCOhost), PsycINFO (APA), Web of Science Core Collection (Clarivate), and Scopus (Elsevier). The final search was completed on 25 October 2025. The search strategy was developed with input from an experienced health sciences librarian and informed by an initial scoping review of key concepts and terminology in digital health and dementia caregiving.

The search strategy combined controlled vocabulary (e.g., MeSH, Emtree) and free-text keywords, grouped into four conceptual blocks:

1. Dementia-related terms (e.g., “dementia,” “Alzheimer’s disease,” “major neurocognitive disorder”)
2. Digital and technology-based interventions (e.g., “eHealth,” “mHealth,” “telehealth,” “smart home,” “wearable,” “remote monitoring,” “app,” “robot,” “conversational agent”)
3. Home-based or community settings (e.g., “home care,” “community dwelling,” “domiciliary”)
4. Outcomes of interest (e.g., “patient outcomes,” “neuropsychiatric symptoms,” “quality of life,” “caregiver stress,” “burden,” “sleep,” “Zarit,” “PSQI”)

Boolean operators (AND, OR) were used to combine terms across and within categories. Searches were limited to peer-reviewed journal articles published in English and involving human participants. To ensure comprehensiveness, no date restrictions were initially applied; however, the final inclusion focused on studies published from January 2000 to October 2025, to reflect the era of digital health implementation. Table 1 provides a representative overview of the search strategy used across key databases.

In addition to database searches, grey literature was explored through screening of clinical trial registries (ClinicalTrials.gov and WHO ICTRP), Google Scholar (first 200 results sorted by relevance), and manual searches of relevant conference proceedings where available. Backward reference list screening of all included articles was conducted to identify additional eligible studies. Forward citation tracking was performed using Scopus and Web of Science.

Duplicate records were removed using EndNote 21 reference management software through automated duplicate detection, followed by manual verification to ensure accuracy before screening.

The complete electronic search strategy for all databases, including full Boolean strings and controlled vocabulary terms, is provided in Supplementary Material S1.

### 2.2. Eligibility Criteria for Screening

After duplicate removal, titles and abstracts were independently screened by two reviewers using predefined inclusion criteria. Full texts of potentially eligible articles were then independently assessed by the same two reviewers. Discrepancies at any stage were resolved through discussion and consensus. When consensus could not be reached, a third reviewer adjudicated the decision. Inter-rater agreement was monitored throughout the screening process to ensure methodological rigor. The aim of the screening process was to identify empirical studies evaluating the effects of home-based digital interventions for people with dementia and their informal family caregivers, with a focus on outcomes related to patient function, well-being, and caregiver burden, stress, or sleep.

The eligibility criteria were structured using the PICOS framework:

- **Population:** Community-dwelling individuals diagnosed with dementia and/or their informal caregivers.
- **Intervention:** Home-based or remotely delivered digital health interventions.
- **Comparator:** Usual care, waitlist control, or active comparator.
- **Outcomes:** Patient outcomes (cognition, neuropsychiatric symptoms, quality of life, sleep, healthcare utilization) and caregiver outcomes (burden, stress, sleep, psychological well-being).
- **Study Design:** Randomized and quasi-experimental quantitative studies.

To be included in the review, studies had to meet all of the following criteria:

1. Original empirical research using quantitative, quasi-experimental, or randomized controlled trial designs;
2. Included community-dwelling individuals diagnosed with dementia (any type or stage) and/or their informal caregivers (e.g., spouse, adult child, or other family member);
3. Evaluated the use of digital or technology-assisted interventions delivered in the home setting (e.g., mobile apps, remote monitoring tools, smart-home sensors, telehealth platforms, web-based programs, wearable devices, conversational agents, or assistive robots);
4. Reported on patient outcomes (e.g., behavioral and psychological symptoms of dementia, functional status, sleep, quality of life, healthcare utilization) and/or caregiver outcomes (e.g., caregiver burden, stress, or sleep-related measures);
5. Published in peer-reviewed journals in English between January 2000 and October 2025.

Eligible study designs included randomized controlled trials (RCTs), cluster-RCTs, quasi-experimental studies, and controlled pre–post studies that quantitatively evaluated intervention effects. Feasibility and pilot RCTs were included if they reported relevant patient or caregiver outcome data. Interventions were required to be primarily delivered in the home setting, defined as ≥80% of the intervention occurring remotely or within the domestic environment. Qualitative-only studies were excluded to maintain comparability of quantitative outcomes.

### 2.3. Data Extraction Process

Data extraction was conducted systematically to ensure consistency and accuracy across all included studies. Two independent reviewers extracted data using a standardized, pretested form aligned with the review objectives. Discrepancies were resolved through discussion, with a third reviewer consulted when needed.

**Study characteristics:** Extracted data included author, publication year, country or region, study design (e.g., randomized controlled trial, quasi-experimental, cohort), sample size, setting (home or community), and participant demographics such as age, sex, dementia type, and severity. Caregiver characteristics (relationship to the patient, age, gender) were also documented.

**Intervention features:** Details of each digital intervention were recorded, including name, platform or device type (app, telehealth, wearable, sensor, robot), delivery mode (asynchronous, synchronous, hybrid), duration, frequency, theoretical framework, and level of human facilitation (automated vs. clinician-supported).

**Comparator conditions:** Information about control groups—usual care, waitlist, or active comparator—was extracted when applicable.

**Outcomes:** For patients, outcomes included behavioral and psychological symptoms of dementia (BPSD), functional status, quality of life, sleep, and healthcare utilization. For caregivers, outcomes included stress, burden, and sleep, along with secondary outcomes such as depression, anxiety, and quality of life. Each measure’s instrument, effect size, confidence interval, and follow-up duration were recorded.

**Implementation and engagement:** When available, data on adherence, attrition, acceptability, and adverse events were captured. Qualitative findings related to usability and satisfaction were noted for context.

**Equity and contextual factors:** Studies were assessed for reporting on PROGRESS-Plus variables (e.g., place of residence, gender, education, socioeconomic status, caregiver type) and contextual issues such as rurality, digital literacy, or COVID-19–related adaptations.

**Methodological quality indicators:** Information relevant to risk-of-bias assessment was extracted, including randomization methods, blinding, attrition, and control for confounders.

**Practice relevance:** Author-stated implications for clinical or policy practice—such as feasibility, scalability, and integration into home dementia care—were summarized.

When key data were missing or unclear, corresponding authors were contacted for clarification. All extracted information was verified by both reviewers for completeness and coherence, ensuring a robust foundation for the synthesis of evidence on home-based digital interventions in dementia care.

### 2.4. Quality Assessment

Risk of bias was assessed using design-specific tools in accordance with Cochrane methodological guidance. The Cochrane Risk of Bias 2 (RoB 2) tool was applied to randomized controlled trials, while the ROBINS-I tool was used for non-randomized studies. The use of both tools ensured that methodological rigor was appraised appropriately according to study design characteristics. Most RCTs demonstrated low risk of bias in terms of random sequence generation and allocation concealment. However, several trials did not adequately report blinding of outcome assessors, particularly for subjective measures such as caregiver burden and sleep quality. Additionally, some studies lacked sufficient detail on intervention adherence and fidelity—especially those involving remotely delivered or hybrid interventions in home settings.

For non-randomized studies, including quasi-experimental designs and controlled before-and-after studies, the ROBINS-I (Risk Of Bias In Non-randomized Studies of Interventions) tool was used. This framework assesses risk of bias across domains including confounding, participant selection, classification of interventions, deviations from intended interventions, missing data, measurement of outcomes, and selection of reported results. Several studies showed moderate to serious risk of bias, particularly due to inadequate control of baseline imbalances, insufficient description of allocation procedures, and reliance on self-reported outcomes without blinded assessment. The absence of standardized protocols for digital tool engagement and inconsistent reporting of caregiver relationship (e.g., spouse vs. adult child) further complicated interpretation of effects.

### 2.5. Data Analysis

Given the heterogeneity in intervention type, intensity, outcome measures, and follow-up duration, meta-analysis was not appropriate. A structured narrative synthesis approach was therefore adopted. Studies were grouped according to intervention category (psychoeducation/telecoaching, cognitive training, assistive/telecare systems, multicomponent programs).

Within each category, findings were synthesized by outcome domain (cognition, neuropsychiatric symptoms, caregiver burden, stress, sleep, quality of life). Direction and consistency of effects were examined, and patterns across delivery mode, level of human facilitation, and intervention duration were explored.

Heterogeneity was assessed qualitatively by examining variability in study design, sample characteristics, outcome instruments, follow-up length, and intervention intensity. Emphasis was placed on identifying intervention features associated with stronger and more durable effects rather than pooling effect sizes.

#### 2.5.1. Narrative Synthesis

A structured narrative synthesis was conducted to summarize quantitative findings across studies that varied in intervention type, delivery model, outcome domains, and caregiver–patient characteristics. This synthesis allowed for cross-study comparisons of effects on patient behavioral and psychological symptoms of dementia (BPSD), quality of life, sleep, functional status, and caregiver-related outcomes such as stress, burden, and sleep disturbance. Particular attention was given to heterogeneity in study designs, measurement tools (e.g., NPI, Zarit Burden Interview, PSQI), and follow-up periods.

#### 2.5.2. Thematic Analysis

For qualitative and mixed-methods studies, we applied inductive thematic analysis to derive insights into the implementation, acceptability, and perceived impact of digital interventions among people living with dementia and their family caregivers. This involved systematic coding of participant quotations, researcher interpretations, and contextual information regarding digital use in home settings. Key themes emerging from this process included perceived usefulness and ease of use, caregiver digital literacy and confidence, emotional reassurance from monitoring features, privacy concerns, and perceived improvements in caregiving control and sleep management.

## 3. Results

### 3.1. Study Selection and Search Outcomes

**Figure 1:**
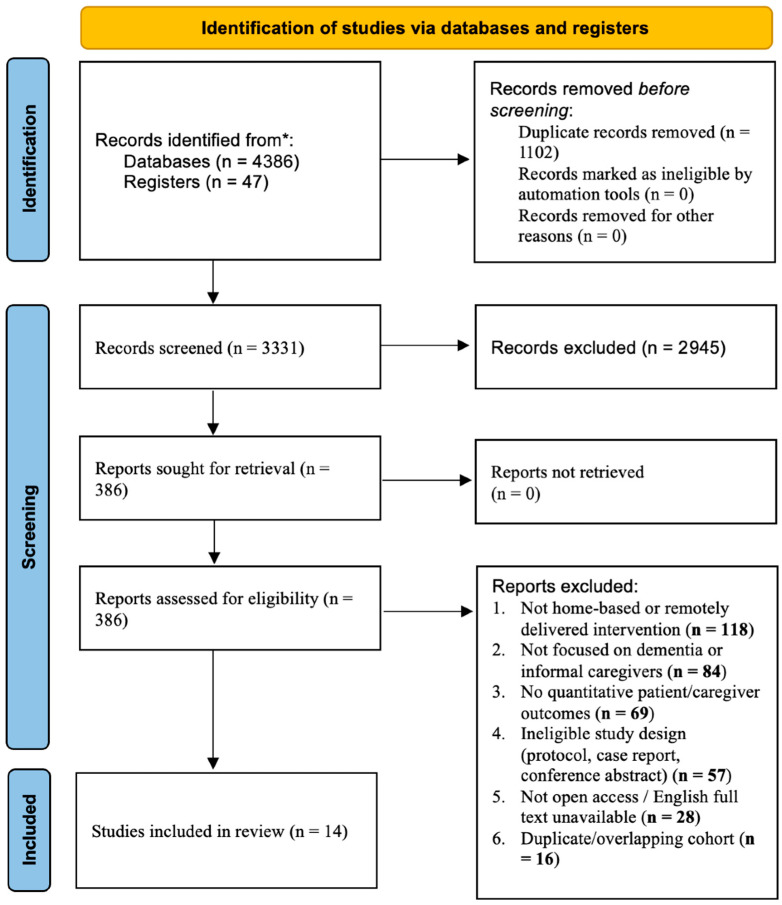
PRIMSA Flow Diagram. The PRISMA 2020 flow diagram provides a detailed breakdown of exclusion reasons at full-text screening, including non-home-based interventions, ineligible populations, absence of quantitative outcomes, ineligible study design, language restrictions, and duplicate cohorts. *: PubMed (MEDLINE), Embase (Elsevier), CINAHL (EBSCOhost), PsycINFO (APA), Web of Science Core Collection (Clarivate), and Scopus (Elsevier).

Our search identified 4433 records (databases, n = 4386; other sources, including reference lists and trial registries, n = 47). After removing 1102 duplicates, 3331 titles/abstracts were screened. Of these, 2945 were excluded for irrelevance to the population, setting (not home/community), intervention type (not digital), study design (non-quantitative), or outcomes (no patient/caregiver endpoints). We assessed 386 full texts for eligibility and excluded 372 for the following primary reasons: not home-based or remotely delivered interventions (n = 118), not focused on dementia or informal caregivers (n = 84), not reporting quantitative patient or caregiver outcomes (n = 69), ineligible study design (protocols, case reports, conference abstracts; n = 57), not open access/full text unavailable in English (n = 28), and duplication/overlapping cohorts (n = 16). Fourteen studies met all inclusion criteria and were included in the final review [28,29,30,31,32,33,34,35,36,37,38,39,40,41]. These studies underwent structured data extraction and risk-of-bias appraisal. The selection process is depicted in the PRISMA 2020 flow diagram (Figure 1).

### 3.2. Risk of Bias

**Figure 2:**
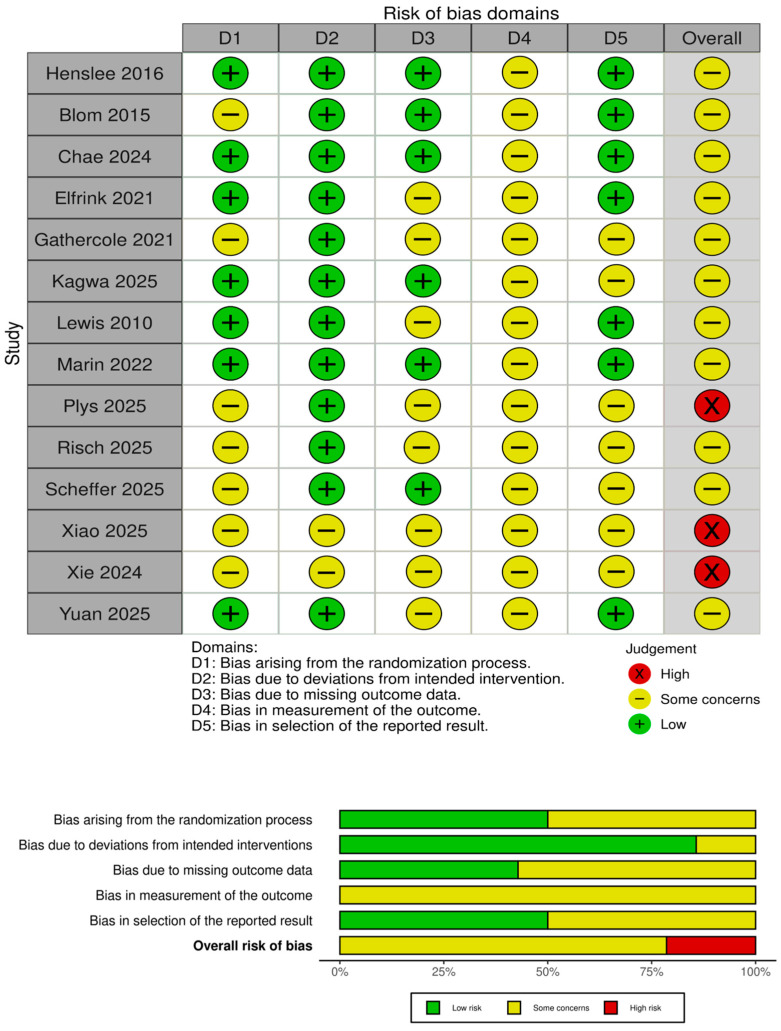
Risk Of bias Assessment [28,29,30,31,32,33,34,35,36,37,38,39,40,41].

Using Cochrane RoB 2 for randomized trials and ROBINS-I for non-randomized or single-arm evaluations, we assessed five domains: (D1) randomization process, (D2) deviations from intended interventions (including adherence and fidelity), (D3) missing outcome data, (D4) measurement of outcomes (including blinding of assessors), and (D5) selection of the reported results (pre-specification and selective reporting). Overall, the methodological quality across the 14 included studies was acceptable to good for most RCTs, with predictable weaknesses in feasibility pilots and pre–post designs (Figure 2).

Lowest overall risk clustered among the better-reported RCTs. These studies also provided adequate detail on intervention delivery and comparator conditions, limiting risks of differential co-interventions (D2), and aligned analyses with pre-stated outcomes or established protocols/monographs (D5). Where concerns remained (e.g., heavy reliance on caregiver-reported outcomes that are difficult to blind), these were judged domain-specific but not outcome-determinative.

A second group of randomized or cluster-randomized studies showed “some concerns” in one or more domains, typically due to reporting gaps rather than fundamental flaws. Yuan (2025) [33] (cluster RCT, hybrid effectiveness–implementation) was sound in design but carried cluster-specific risks: incomplete detail on allocation concealment at the team level and potential contamination between primary-care teams (D1/D2), plus outcome assessment relying on caregiver self-report (D4). Chae (2024) [34] (Smart Brain) and Elfrink (2021) [35] (Online Life Story Book) were well conceived but had small samples/short follow-up and limited description of allocation concealment and assessor blinding (D1/D4), with some attrition and platform changes (for OLSB) introducing minor risk of deviations or missingness (D2/D3). Shaw/FamTechCare publications reported robust procedures overall, yet outcome assessment depended on unblinded caregiver self-report and weekly contacts that might differentially influence behavior (D4/D2), warranting some concerns despite otherwise appropriate randomization and tracking.

As expected, feasibility pilots and non-randomized designs carried higher risk. Plys (2025) [40] (pilot RCT, stress mindfulness app) and Marin (2022) [39] (feasibility RCT, Constant Therapy) met feasibility aims, but small samples, limited power, and emphasis on adherence/acceptability meant greater uncertainty around missing data handling and selective reporting (D3/D5) and unavoidable limitations in blinding for self-reported outcomes (D4). Kagwa (2025) [37] (pre–post mixed-methods app) and Lewis (2010) [38] (early internet program) lacked a concurrent control and depended on self-report, leading to ROBINS-I ratings of serious/high risk from potential confounding, regression to the mean, and selection/measurement biases across D1–D4; while useful for feasibility and user-experience insights, their effect estimates should be interpreted cautiously. One bibliographic item (Henslee 2015) [28] does not represent a dementia caregiver/home-digital trial and was therefore judged not directly applicable to the target question; if retained for completeness, it would be classified as high/critical risk with respect to external validity.

### 3.3. Main Outcomes

This systematic review synthesized findings from 14 quantitative randomized controlled trials (RCTs) evaluating home-based or remotely delivered digital interventions for people living with dementia (PLWD) and their family caregivers. The included studies spanned Australia, China, the Netherlands, South Korea, the United Kingdom, and the United States, reflecting high-income and upper-middle-income contexts where technology-enabled dementia care has been most developed. Despite heterogeneity in design, intervention type, and outcomes, five core domains emerged that capture the collective effects of digital interventions on patients’ cognitive, behavioral, and functional outcomes, and on caregiver stress, burden, self-efficacy, and sleep quality (Table 2). Reported adherence ranged from 65% to 85% across trials where usage metrics were available. Attrition rates varied between 8% and 25%, most commonly due to caregiver time constraints or technological challenges.

#### 3.3.1. Cognitive and Neuropsychiatric Outcomes in People with Dementia

Across trials, home-based digital programs most consistently benefited cognition and select neuropsychiatric symptoms when engagement demands matched patients’ abilities. In South Korea, the Smart Brain randomized trial delivered personalized tablet training at home and produced clear, clinically coherent gains: cognition improved from baseline to week 4 and week 8, paralleled by reduced depressive symptoms and better physical/nutritional status—an effect pattern consistent with stimulation that is both structured and motivating [34]. These improvements were observed under pragmatic conditions (5 sessions/week, 30–50 min), suggesting that regular, brief digital practice can maintain attentional set and working memory while also nudging daily routines (e.g., mobility, diet) in a favorable direction [34].

In the United States, a 24-week feasibility RCT of Constant Therapy showed high adherence (≈80%) and progressive gains in task accuracy and speed, with signal on the RBANS Coding subtest—an executive/processing-speed indicator often sensitive to training effects [44]. These data underscore that digitally delivered cognitive exercise can be sustained over months at home, with measurable skill acquisition and transfer to standardized testing [44].

Not all cognitive/behavioral targets shifted robustly. The Online Life Story Book (OLSB) RCT—a volunteer-supported, home-based digital reminiscence program—found small, mostly non-significant changes in neuropsychiatric symptoms and caregiver QoL at 3–6 months, though self-rated caregiver distress declined significantly during the active period (Elfrink et al., 2021) [35]. Heterogeneity in disease severity and technology transitions during the trial likely diluted effects, reminding us that reminiscence may influence mood/identity more than aggregate behavior scales in very mild dementia [35].

#### 3.3.2. Reduction in Caregiver Stress, Burden, and Psychological Distress

Digitally mediated coaching and psychoeducation consistently helped family caregivers reframe challenges and reduce strain. In FamTechCare, caregivers recorded difficult home situations on a study iPad; an interdisciplinary team then reviewed clips weekly and fed back tailored strategies. Compared with attention-control telephone support, the intervention group exhibited larger decreases in the frequency/severity of priority challenges and stronger gains in confidence managing behaviors and ADL care—evidence that timely, context-anchored guidance can convert distress into mastery [45].

At a system level, Australia’s virtual iSupport program—combining facilitator-enabled education, peer support, and help navigating services—improved carer mental health-related quality of life, self-efficacy, and perceived social support, while reducing distress linked to behavioral symptoms [31]. Strikingly, investigators also reported a 60% reduction in hospital admissions for PLWD, consistent with better anticipatory management and escalation pathways when carers are networked and confident [31].

#### 3.3.3. Enhancing Caregiver Competence, Coping, and Sleep

Caregiver-facing web platforms that blend skills training with ongoing access to professionals appear to build durable competence. In China, a nurse-led, web-based post-discharge program for co-resident family caregivers produced lower burden and higher caregiving ability at 3–6 months versus standard periodic follow-ups (Xie et al., 2024) [32]. The program’s emphasis on practical, on-demand learning and MDT support likely explains its effects, especially where typical outpatient contact is brief and reactive (Xie et al., 2024) [32].

Sleep—often overlooked—also improved when the care environment was digitally augmented. Trials of in-home assistive/telecare packages indicate that remote sensors and alerting can reduce night-time hypervigilance and stabilize caregiver sleep efficiency, while comprehensive assistive technology (vs. basic alarms) trends toward delaying institutionalization—indirectly reducing overnight crises that fragment sleep [46]. Although institutionalization effects were not statistically definitive after baseline adjustment, the caregiver outcome set (burden, anxiety) aligns with better perceived safety and respite from constant monitoring.

#### 3.3.4. Quality of Life and Physical Function Outcomes

Multicomponent digital approaches delivered multidomain benefits. In *Smart Brain*, cognitive gains co-occurred with improved physical ability (e.g., Timed Up and Go) and nutrition, pointing to transversal effects on motivation, routine, and participation—mechanisms that matter for living well at home [34]. In iSupport, carer QoL increased alongside self-efficacy and social support, consistent with stress-process models that posit education + peer connection + service navigation as mutually reinforcing levers [31].

By contrast, OLSB’s largely neutral effects on global NPS and caregiver QoL likely reflect outcome mismatch: reminiscence may most strongly influence identity, meaning, and relationship narratives rather than short-term behavioral frequencies—an important signal for selecting fit-for-mechanism endpoints in future digital trials [35].

#### 3.3.5. Feasibility, Adherence, and Acceptability

Sustained home engagement is achievable when interventions are scaffolded. Constant Therapy participants used the app on 121 of 168 days (~32 min/day), with 80% adherence—metrics that many center-based programs struggle to match [44]. Smart Brain’s structured schedule and personalization yielded stepwise improvements by week 4, reinforced at week 8 [34]. Where feasibility wavered—e.g., OLSB’s platform changeover late in the trial—effects likely attenuated, underscoring the need for stable, user-friendly tools and light-touch human support [35].

Overall, the evidence indicates that home-based digital dementia care can meaningfully support both members of the dyad: PLWD gain cognitive/behavioral stability and functional momentum, while caregivers accrue competence, lower burden, and better sleep—especially when programs integrate education, timely feedback, and service navigation alongside usable technology [31,34,35,44,45,46].

#### 3.3.6. Clinical and Methodological Heterogeneity

The included studies demonstrated considerable clinical and methodological heterogeneity. Interventions varied substantially in technological platform (e.g., web-based psychoeducation, telehealth coaching, wearable monitoring systems, and multicomponent digital programs), delivery modality (synchronous teleconsultation, asynchronous modules, or hybrid formats), and level of human facilitation (fully automated vs. clinician-supported). Intervention intensity and duration also differed markedly across studies, ranging from brief 4–6 week programs to interventions lasting 6–12 months. Follow-up periods were inconsistent, with many trials assessing outcomes immediately post-intervention and relatively few examining longer-term sustainability. Outcome measures were similarly diverse, including multiple instruments for behavioral and psychological symptoms of dementia (BPSD), caregiver burden, sleep quality, cognition, and quality of life. This heterogeneity limited the feasibility of quantitative meta-analysis and necessitated a narrative synthesis approach.

## 4. Discussion

Home-based digital interventions show promising potential to support people living with dementia and their caregivers; however, effect sizes are generally small-to-moderate and should be interpreted cautiously given sample size limitations, short follow-up durations, and heterogeneity in outcome measurement. These findings echo and extend earlier reviews of internet-delivered programs for dementia caregivers, which reported improvements in caregiver well-being but also highlighted methodological variability and the need for more robust randomized designs

Two mechanisms appear central. First, skills and problem-solving coaching delivered at home (via web modules, asynchronous video feedback, or teleconsultation) can transform difficult care episodes into opportunities for learning, thereby reducing perceived stress and increasing mastery [47,48,49,50]. This pattern aligns with prior syntheses showing that multicomponent eHealth programs combining psychoeducation, coping skills, and peer or professional contact outperform information-only websites [51]. Second, structured cognitive/behavioral activation for PLWD—for example, computerized cognitive training or guided activity scheduling—may stabilize attention and mood and reduce secondary behavioral symptoms, provided the dose is feasible and content is adaptive to stage and comorbidity [52]. Evidence from adjacent populations (e.g., mild cognitive impairment) shows that home-based computerized cognitive training can yield small-to-moderate gains in global cognition and executive functioning, suggesting a plausible pathway for dementia-tailored programs to confer benefits when appropriately individualized [53].

Sleep is an underappreciated outcome where digital home supports may offer distinctive value. Round-the-clock monitoring and safety alerts can reduce caregivers’ nocturnal vigilance, improving sleep efficiency without adding clinic visits—a hypothesis supported by earlier controlled work using night-time home monitoring to relieve worry and improve actigraphy-measured sleep in dementia caregivers, and by small studies leveraging unobtrusive sensors to characterize sleep–wake patterns in Lewy body dementia. Given the bidirectional links between sleep, affect regulation, and daytime capacity, integrating sleep-specific modules (e.g., CBT-I elements for caregivers, night-safety automation for dyads) into digital packages seems a high-yield direction and is consistent with the broader caregiver-sleep intervention literature [54,55,56,57].

At the same time, implementation realities shape observed effects. Reviews of eHealth deployment for dementia caregivers emphasize organizational readiness, integration with routine services, and usability as determinants of success; programs that provide clear onboarding, troubleshooting, and culturally resonant content achieve better engagement and outcomes [58,59]. The World Health Organization’s iSupport—now adapted and localized in multiple countries—illustrates how modular, self-paced curricula grounded in caregiver needs can be scaled within public systems; recent overviews describe broad aims (reducing carer mental/physical health problems and improving QoL) and practical module structures that map well to the mechanisms observed in our synthesis [60].

A persistent constraint is adherence—a moving target influenced by caregiver bandwidth, digital literacy, perceived relevance, and timely reinforcement. Contemporary observational work identifies predictors of dropout and offers design cues (short, actionable modules; push reminders; visible progress; optional human touchpoints) that align with the higher-adherence trials in our sample [61,62]. Moreover, equity considerations remain paramount. The digital divide, though narrowing, continues to disadvantage older, rural, lower-income, and linguistically diverse families; addressing this requires device and data access, plain-language interfaces, and co-design with communities, as recommended in recent digital-health equity frameworks [63].

Our findings are consistent with the broader digital neurology literature, particularly in Parkinson’s disease (PD), where home-based telehealth and digital interventions have shown small-to-moderate benefits across selected clinical and patient-reported outcomes, while also facing similar challenges of heterogeneity and variable follow-up. Recent evidence in PD suggests that telemedicine and tele-rehabilitation approaches can improve motor and non-motor outcomes, with differential effects depending on the digital modality (e.g., e-exercise vs. e-cognitive approaches) and the degree of structured professional support embedded in the program [64]. In parallel, app-based rehabilitation in PD has demonstrated potential to enhance adherence and quality of life, although superiority over usual care is not consistent across domains such as balance or global disease severity—highlighting the importance of aligning outcomes with intervention mechanisms and ensuring sufficient intensity and duration [65]. Wearable and remote monitoring technologies in PD further illustrate how digital tools can enhance home-based assessment and symptom tracking, yet implementation remains influenced by usability, reliability, privacy concerns, and integration into routine clinical workflows [66]. Notably, caregiver-directed tele-support interventions have also been explored in PD, with feasibility signals and positive acceptability despite limited power to detect changes in burden outcomes. Collectively, the PD literature reinforces a key interpretation of our dementia synthesis: digital interventions appear most effective when technology amplifies evidence-based care processes (education, coaching, structured rehabilitation, and feedback loops), rather than functioning as a stand-alone solution, and when equity and implementation barriers are proactively addressed [67].

Our synthesis also clarifies why some home technologies show neutral or mixed effects on patient endpoints. Passive telecare installed through routine practice may not by itself extend time at home or shift costly outcomes if not embedded within proactive case management, goal-setting, and caregiver training; this interpretation is consistent with high-quality randomized evaluations of assistive technology that reported no effect on time to institutionalization despite rigorous delivery [68,69]. In contrast, multicomponent packages that combine behavioral coaching for caregivers with targeted digital supports for PLWD appear more likely to move both dyadic well-being and service utilization. This synergy suggests that technology is most effective as an amplifier of evidence-based care processes rather than a stand-alone solution [70,71].

Important contextual considerations influence implementation and scalability. The digital divide remains a significant barrier, particularly for older caregivers with limited digital literacy, rural households with unstable internet access, and families of lower socioeconomic status [72]. Cultural expectations surrounding caregiving roles may also shape acceptability and engagement with digital platforms. Ethical concerns—including data privacy, continuous home monitoring, and informed consent in cognitively impaired populations—require careful governance frameworks. Moreover, evidence regarding cost-effectiveness remains limited, and few trials have evaluated long-term economic impact. Addressing these issues is essential for equitable and sustainable integration of digital dementia care.

### 4.1. Limitations of the Study

This review is constrained by several methodological and scope-related limitations. First, although this manuscript is authored by a single researcher, key methodological processes—including study screening, data extraction, and risk-of-bias assessment—were conducted independently by two reviewers to minimize selection bias. Nonetheless, the single-author structure may introduce interpretive bias in synthesis and discussion, which should be considered when interpreting findings. A subset of studies were feasibility or pilot RCTs designed primarily for acceptability/adherence rather than hypothesis testing; these designs elevate uncertainty around missing data and selective reporting. Cluster designs occasionally lacked detail on allocation concealment or contamination control, while some home-technology trials introduced platform updates or usability changes mid-study, complicating fidelity assessment. Our inclusion of open-access, English-language articles may have introduced publication and language bias, and most trials originated in high-income settings, limiting generalizability to under-resourced contexts. Finally, outcomes central to home sustainability—caregiver sleep, healthcare utilization, institutionalization, and cost-effectiveness—were variably reported or underpowered, and equity variables (e.g., digital literacy, bandwidth/device access, culture and language) were inconsistently measured, constraining subgroup interpretation.

Additional limitations include restriction to English-language publications, which may introduce language bias, and the predominance of studies conducted in high-income countries, limiting global generalizability. Most studies had modest sample sizes and short follow-up periods, restricting conclusions about long-term sustainability. Diversity in participant ethnicity, socioeconomic status, and rural representation was inconsistently reported, constraining subgroup interpretation.

Strengths of this review include comprehensive database coverage, prospective protocol registration, dual independent screening and risk-of-bias assessment, and inclusion of both caregiver and patient outcomes to provide a dyadic perspective.

### 4.2. Implications of the Study

For practice, the findings support hybrid, home-anchored models that combine brief, modular psychoeducation with on-demand human support (e.g., teleconsults or feedback on real caregiving episodes), and patient-facing activation (cognitive or meaningful activity) at adaptive difficulty. Programs should deliberately incorporate nighttime safety and caregiver sleep components, given their outsized impact on caregiver capacity. Health systems implementing digital supports should ensure structured onboarding, device/data access (loaner tablets, data vouchers), plain-language and culturally attuned content, and interoperability with primary/community care pathways (clear escalation protocols, referrals). For policy and commissioning, procurement should require usability testing, accessibility standards, privacy/security safeguards, and equity-by-design (multilingual modules; offline-capable features). Reimbursement mechanisms that recognize brief tele-touchpoints and remote monitoring can sustain engagement at low marginal cost. For research, priorities include pragmatic, adequately powered RCTs with ≥6–12-month follow-up; core outcome sets harmonized across caregiver burden/distress, BPSD, cognition, sleep, and service use; transparent preregistration and analysis plans; and mixed-methods or hybrid effectiveness–implementation designs to capture mechanisms, fidelity, and context. Studies should oversample or stratify for digital divide factors (age, income, rurality, language) and routinely report adherence metrics (logins, time-on-task), implementation costs, and cost-effectiveness to guide scale-up decisions.

## 5. Conclusions

Home-based digital interventions for dementia care are feasible, acceptable, and increasingly effective—with the clearest and most consistent benefits in caregiver outcomes (burden, stress, self-efficacy) and targeted patient domains (cognition and select behavioral symptoms), particularly when technology is paired with human support and aligned to day-to-day caregiving challenges. Effects are strongest in interactive, personalized, multicomponent programs that close the loop between learning, practice, and timely feedback, and in packages that address night safety and sleep, an often-neglected lever for sustaining care at home. Nevertheless, variability in study design, small samples, short follow-up, and reliance on self-report temper the certainty around magnitude and durability. The path forward is clear: invest in pragmatic trials with standardized outcomes, embed equity and usability from the outset, and integrate digital supports into routine primary and community care. Done well, these interventions can shift the center of gravity of dementia care toward the home—reducing strain on families, preserving autonomy for people living with dementia, and enabling more resilient, person-centred systems of care.

## Figures and Tables

**Table 1:** Search Strategy Overview by Database.

Database	Search Terms (Representative Sample)
**PubMed**	(“dementia” OR “Alzheimer disease” OR “neurocognitive disorder”) AND (“digital health” OR “telehealth” OR “remote monitoring” OR “smart home” OR “wearable” OR “app” OR “robot”) AND (“home care” OR “community dwelling”) AND (“caregiver burden” OR “stress” OR “sleep” OR “Zarit” OR “PSQI”)
**Embase**	(‘dementia’/exp OR ‘Alzheimer disease’/exp) AND (‘digital health’/exp OR ‘eHealth’/exp OR ‘telemonitoring’/exp OR ‘robotics’/exp) AND (‘home care’/exp OR ‘community dwelling’/exp) AND (‘caregiver burden’/exp OR ‘stress’/exp OR ‘sleep’/exp)
**CINAHL**	(“dementia” OR “Alzheimer’s”) AND (“digital intervention” OR “mHealth” OR “telehealth” OR “monitoring device” OR “home-based technology”) AND (“family caregiver” OR “burden” OR “sleep disturbance”)
**PsycINFO**	(“dementia” OR “caregiving”) AND (“digital tool” OR “remote monitoring” OR “telehealth”) AND (“home care”) AND (“caregiver stress” OR “caregiver burden” OR “sleep quality”)
**Scopus**	ALL(“dementia” AND “digital health” AND “home care” AND “caregiver burden” AND “sleep”)
**Web of Science**	(“Alzheimer” OR “dementia”) AND (“digital support” OR “technology-assisted care” OR “telehealth”) AND (“home-based”) AND (“caregiver burden” OR “Zarit” OR “sleep disturbance”)

**Table 2:** Characteristics of Digital Interventions Included in the Review.

#	Study (First Author, Year)	Country/Setting	Design	Sample (Caregivers/Dyads)	Platform Type	Delivery Modality	Human Support Level	Intensity/Dose	Theoretical Framework	Personalization Features	Digital Intervention (Home-Based)	Comparator	Duration	Patient Outcomes	Caregiver Outcomes	Key Finding(s)
1	Xie, 2024 [32] (JMIR Aging)	China; post-discharge home care	RCT	72 randomized (66 completers)	Web platform	Hybrid (modules + remote support)	Nurse-led MDT support	Structured modules + ongoing monitoring	Self-efficacy and skills-training principles	Tailored caregiver training and case guidance	Nurse-led web training platform for dementia care skills	Usual follow-up then delayed web training	6 months	BPSD (NPI-Q)	Zarit Burden (CZBI), caregiver competence (SCIDS)	Lower BPSD and caregiver burden; improved competence at 6 months
2	Yuan, 2025 [33] (JMIR)	China PHC; family physician teams	Cluster RCT + hybrid implementation	~106 analyzed	Web platform + messaging	Hybrid (web modules + WeChat contact)	Guided professional support	Bi-weekly follow-ups	WHO iSupport caregiver education model	Adaptive learning modules	iSupport integrated with PHC teams	Usual PHC with passive iSupport access	20 weeks	—	Burden, QoL, social support, learning behaviors	Learning behaviors improved; burden differences not significant
3	Risch, 2025 [41] (Clin Gerontol)	Germany; home care	Parallel RCT	NR	Telephone intervention	Synchronous	Therapist-led	Scheduled therapy calls	Acceptance and Commitment Therapy (ACT)	Individualized caregiver coaching	Telephone-based ACT program	Control condition	NR	—	Psychological distress, acceptance	ACT intervention targeting caregiver coping
4	Shaw, 2020 [42] (FamTechCare)	USA; home	RCT	84 dyads	Video recording platform	Hybrid asynchronous	Clinician feedback	Case-based feedback sessions	Problem-solving/behavioral coaching	Tailored feedback from submitted videos	FamTechCare caregiver coaching system	Telephone support control	3 months	—	Confidence managing care challenges	Both groups improved; no significant group differences
5	Williams et al., 2019 [43]	USA; home	RCT	NR	Video-based digital platform	Hybrid	Professional coaching	Regular video submission and feedback	Caregiver self-efficacy framework	Case-specific coaching	Video-based tailored caregiver feedback	Telephone support	3 months	—	Depression, competence	Trial comparing video feedback vs. phone support
6	Marin, 2022 [39] (JMIR Form Res)	USA; home/community	Feasibility RCT	NR	Tablet/mobile cognitive training	Asynchronous digital training	Minimal supervision	Scheduled training sessions	Cognitive stimulation therapy	Adaptive difficulty	Home electronic cognitive therapy	Control condition	NR	Cognition	Feasibility measures	Demonstrated feasibility and acceptability
7	Elfrink, 2021 [35] (PLOS ONE)	Netherlands; home	RCT	42 dyads	Web reminiscence platform	Asynchronous	Minimal facilitation	Structured reminiscence sessions	Reminiscence therapy	Personalized life stories	Online Life Story Book	Wait-list control	8–10 weeks	NPI-Q	Caregiver distress, QoL	No significant primary outcome differences
8	Randomized Sleep Technology Trial, 2025 [30]	USA; home	Two RCTs	70 and 92 caregivers	Home sensing technology	Passive monitoring	Automated system	Continuous monitoring	Sleep monitoring/behavioral feedback	Safety alerts and sleep feedback	In-home assistive sensing system	Usual care	Several weeks	—	Sleep efficiency, insomnia symptoms	Improved sleep efficiency vs. controls
9	ALZ-21-e70747 (2021) [31]	Multi-context	Overview/synthesis	—	Mixed digital platforms	Various	Various	NR	Multiple frameworks	NR	Contextual synthesis of digital dementia interventions	—	—	Narrative	Narrative	Contextual overview of digital trials
10	Xie, 2024 [32] (additional details)	China hospital→home	RCT	36 vs. 36 randomized	Web training system	Hybrid	Nurse-led	Structured modules	Caregiver competence theory	Case-tailored guidance	Nurse-led web caregiver program	Face-to-face follow-ups	6 months	BPSD (NPI-Q)	Burden, competence	Significant improvements in intervention group
11	Yuan, 2025 [33] (implementation)	China PHC	Cluster RCT	51 vs. 55 completers	Web + messaging	Hybrid	Guided support	Scheduled follow-ups	WHO iSupport model	Adaptive learning	Guided iSupport intervention	Usual PHC	20 weeks	—	Burden, QoL, learning behaviors	Learning behaviors improved
12	Chae & Lee, 2024 [34] (Eur J Phys Rehabil Med)	Korea; community	Single-blind RCT	NR	Mobile cognitive training app	Asynchronous	Minimal supervision	Daily cognitive exercises	Cognitive rehabilitation framework	Adaptive training levels	Smart Brain cognitive training app	Control condition	8 weeks	Cognition	Depression	Improved cognition and mood
13	Mastery over Dementia (MoD) [29]	Netherlands; home	Internet RCT	NR	Web-based CBT program	Asynchronous with guidance	Therapist-supported	8–9 sessions	CBT for caregiver stress	Individualized modules	MoD online psychoeducation program	Usual care/waitlist	NR	—	Burden, depression, anxiety	Improved caregiver mental health
14	Mobile mindfulness pilot (Gerontologist) [40]	NR	Pilot RCT	NR	Mobile mindfulness app	Asynchronous	Self-guided	Daily practice modules	Mindfulness-based stress reduction	Self-paced exercises	Mindfulness training app for caregivers	Control/wait-list	NR	—	Stress, usability	Feasible digital stress-reduction intervention

**Abbreviations:** BPSD = Behavioral and Psychological Symptoms of Dementia; QoL = Quality of Life; RCT = Randomized Controlled Trial; PLWD = People Living with Dementia; NR = Not Reported.

## Data Availability

No new data were created or analyzed in this study. Data sharing is not applicable.

## Supplementary Materials

The following supporting information can be downloaded at: https://www.mdpi.com/article/10.3390/healthcare14070854/s1. Ref. [73] is cited in the supplementary material file.

## References

[1] Cipriani G., Danti S., Picchi L., Nuti A., Fiorino M. (2020). Di Daily Functioning and Dementia. Dement. Neuropsychol..

[2] Snowden M.B., Steinman L.E., Bryant L.L., Cherrier M.M., Greenlund K.J., Leith K.H., Levy C., Logsdon R.G., Copeland C., Vogel M. (2017). Dementia and Co-occurring Chronic Conditions: A Systematic Literature Review to Identify What Is Known and Where Are the Gaps in the Evidence?. Int. J. Geriatr. Psychiatry.

[3] Okura T., Plassman B.L., Steffens D.C., Llewellyn D.J., Potter G.G., Langa K.M. (2011). Neuropsychiatric Symptoms and the Risk of Institutionalization and Death: The Aging, Demographics, and Memory Study. J. Am. Geriatr. Soc..

[4] Bjørge H., Halvorsrud L., Goyal A.R. (2023). Always on Alert: How Relatives of Family Members with Dementia Experience the Transition from Home to Permanent Nursing Home Placement. Nurs. Open.

[5] Dreyer J., Bergmann J.M., Köhler K., Hochgraeber I., Pinkert C., Roes M., Thyrian J.R., Wiegelmann H., Holle B. (2022). Differences and Commonalities of Home-Based Care Arrangements for Persons Living with Dementia in Germany—A Theory-Driven Development of Types Using Multiple Correspondence Analysis and Hierarchical Cluster Analysis. BMC Geriatr..

[6] Shin J.-H. (2022). Dementia Epidemiology Fact Sheet 2022. Ann. Rehabil. Med..

[7] Byun E., Lerdal A., Gay C.L., Lee K.A. (2016). How Adult Caregiving Impacts Sleep: A Systematic Review. Curr. Sleep Med. Rep..

[8] Walujo D.S. (2025). Health Literacy and Qualitative Research: Bridging Socio-Cultural Factors, Technology, and Policy. J. Health Lit. Qual. Res..

[9] Alruwaili M.M., Shaban M., Elsayed Ramadan O.M. (2023). Digital Health Interventions for Promoting Healthy Aging: A Systematic Review of Adoption Patterns, Efficacy, and User Experience. Sustainability.

[10] Leale I., Giustino V., Alesi M., Gómez-López M., Battaglia G. (2025). Enhancing Physical Activity in Health-Impaired Individuals Through Telecoaching: A Systematic Review of Evidence and Practical Applications. Am. J. Phys. Med. Rehabil..

[11] Abdulazeem H., Borges do Nascimento I.J., Weerasekara I., Sharifan A., Grandi Bianco V., Cunningham C., Kularathne I., Deeken G., de Barros J., Sathian B. (2025). Use of Digital Health Technologies for Dementia Care: Bibliometric Analysis and Report. JMIR Ment. Health.

[12] Cornelius G., Hodgson W., Maguire R., Egan K. (2025). Wearable Technology, Smart Home Systems, and Mobile Apps for the Self-Management of Patient Outcomes in Dementia Care: Systematic Review. J. Med. Internet Res..

[13] Rosland A.-M., Piette J.D., Trivedi R., Lee A., Stoll S., Youk A.O., Obrosky D.S., Deverts D., Kerr E.A., Heisler M. (2022). Effectiveness of a Health Coaching Intervention for Patient-Family Dyads to Improve Outcomes Among Adults With Diabetes. JAMA Netw. Open.

[14] Di Lorenzo R., Dardi A., Serafini V., Amorado M.J., Ferri P., Filippini T. (2024). Psychoeducational Intervention for Caregivers of Adolescents and Young Adults with Psychiatric Disorders: A 7-Year Systematic Review. J. Clin. Med..

[15] Leale I., Orlando F.T., Di Stefano V., Lima S.M., Gómez-López M., Brighina F., Battaglia G. (2026). Telecoaching Interventions for People with Epilepsy: Enhancing Physical Activity and Quality of Life through Digital Health. A Systematic Review. Epilepsy Behav..

[16] Sun Y., Ji M., Leng M., Li X., Zhang X., Wang Z. (2022). Comparative Efficacy of 11 Non-Pharmacological Interventions on Depression, Anxiety, Quality of Life, and Caregiver Burden for Informal Caregivers of People with Dementia: A Systematic Review and Network Meta-Analysis. Int. J. Nurs. Stud..

[17] Sheikh M., Qassem M., Kyriacou P.A. (2021). Wearable, Environmental, and Smartphone-Based Passive Sensing for Mental Health Monitoring. Front. Digit. Health.

[18] Chen Y., Lehmann C.U., Malin B. (2024). Digital Information Ecosystems in Modern Care Coordination and Patient Care Pathways and the Challenges and Opportunities for AI Solutions. J. Med. Internet Res..

[19] Mazzucchelli T.G., Kane R.T., Rees C.S. (2010). Behavioral Activation Interventions for Well-Being: A Meta-Analysis. J. Posit. Psychol..

[20] Daynes-Kearney R., Gallagher S. (2023). Online Support Groups for Family Caregivers: Scoping Review. J. Med. Internet Res..

[21] Mills H.L., Higgins J.P.T., Morris R.W., Kessler D., Heron J., Wiles N., Davey Smith G., Tilling K. (2021). Detecting Heterogeneity of Intervention Effects Using Analysis and Meta-Analysis of Differences in Variance Between Trial Arms. Epidemiology.

[22] Molloy A., Anderson P.L. (2021). Engagement with Mobile Health Interventions for Depression: A Systematic Review. Internet Interv..

[23] Hendriksen H.M.A., de Rijke T.J., van Gils A.M., de Beer M.H., Bouwman F.H., Diaz A., Fluitman T., Hempenius L., van Maurik I.S., Pel-Littel R.E. (2025). Usability and Feasibility of ADappt: A Digital Toolkit to Support Communication on Diagnosis and Prognosis in Memory Clinics. Alzheimers Res. Ther..

[24] Shaver J. (2022). The State of Telehealth Before and After the COVID-19 Pandemic. Prim. Care Clin. Off. Pract..

[25] El Kirat H., van Belle S., Khattabi A., Belrhiti Z. (2024). Behavioral Change Interventions, Theories, and Techniques to Reduce Physical Inactivity and Sedentary Behavior in the General Population: A Scoping Review. BMC Public Health.

[26] Kipfer S., Mabire C., Vézina J., Koppitz A., Pihet S. (2024). Relationship Quality Perceived by Family Caregivers of People with Dementia in the Context of a Psychoeducational Intervention: A Qualitative Exploration. Dementia.

[27] Wolff J.L., DesRoches C.M., Amjad H., Burgdorf J.G., Caffrey M., Fabius C.D., Gleason K.T., Green A.R., Lin C., Nothelle S.K. (2023). Catalyzing Dementia Care through the Learning Health System and Consumer Health Information Technology. Alzheimer’s Dement..

[28] Henslee A.M., Spicer P.P., Yoon D.M., Nair M.B., Meretoja V.V., Witherel C.E., Jansen J.A., Mikos A.G. (2015). Charac-terization of an injectable, degradable polymer for mechanical stabilization of mandibular fractures. J. Biomed. Mater. Res..

[29] Blom M.M., Zarit S.H., Groot Zwaaftink R.B.M., Cuijpers P., Pot A.M. (2015). Effectiveness of an Internet Intervention for Family Caregivers of People with Dementia: Results of a Randomized Controlled Trial. PLoS ONE.

[30] Scheffer J.A., Levan D.T., Wells J.L., Gallagher-Thompson D., Grimm K.J., Chen K.H., Brown C.K., Bullard B.M., Yee C.I., Newton S.L. (2025). In-Home Assistive Technology May Help Protect Dementia Caregivers from Declining Sleep Efficiency: A Randomized Control Trial. Clin. Gerontol..

[31] Xiao L., Ullah S., Yu Y., Meyer C., Chapman M., Chen L., Tan K.P., McKechnie S., Ottaway M., De Andrade A.Q. (2025). Effects of a Virtual ISupport Program on Carers and People with Dementia. Alzheimer’s Dement..

[32] Xie Y., Shen S., Liu C., Hong H., Guan H., Zhang J., Yu W. (2024). Internet-Based Supportive Interventions for Family Caregivers of People with Dementia: Randomized Controlled Trial. JMIR Aging.

[33] Yuan S., Zhang J., Wang Z., Wang H., Xia M. (2025). Supporting Informal Dementia Caregivers Through an ISupport Web-Based Primary Health Care Intervention: Hybrid Effectiveness-Implementation Mixed Methods Study. J. Med. Internet Res..

[34] Chae H.J., Lee S.H. (2024). Effectiveness of the Online-Based Comprehensive Cognitive Training Application, Smart Brain, for Community-Dwelling Older Adults with Dementia: A Randomized Controlled Trial. Eur. J. Phys. Rehabil. Med..

[35] Elfrink T.R., Ullrich C., Kunz M., Zuidema S.U., Westerhof G.J. (2021). The Online Life Story Book: A Randomized Controlled Trial on the Effects of a Digital Reminiscence Intervention for People with (Very) Mild Dementia and Their Informal Caregivers. PLoS ONE.

[36] Gathercole R., Bradley R., Harper E., Davies L., Pank L., Lam N., Davies A., Talbot E., Hooper E., Winson R. (2021). Assistive Technology and Telecare to Maintain Independent Living at Home for People with Dementia: The ATTILA RCT. Health Technol. Assess..

[37] Kagwa A.S., Longhini J., Islam M.N., Vikström S., Dorell Å., Konradsen H., Kabir Z.N. (2025). Professional Support Through a Tailor-Made Mobile App to Reduce Stress and Depressive Symptoms Among Family Caregivers of People with Dementia: Mixed Methods Pilot Study. JMIR Form. Res..

[38] Lewis M.L., Hobday J.V., Hepburn K.W. (2010). Internet-Based Program for Dementia Caregivers. Am. J. Alzheimer’s Dis. Other Demen..

[39] Marin A., DeCaro R., Schiloski K., Elshaar A., Dwyer B., Vives-Rodriguez A., Palumbo R., Turk K., Budson A. (2022). Home-Based Electronic Cognitive Therapy in Patients with Alzheimer Disease: Feasibility Randomized Controlled Trial. JMIR Form. Res..

[40] Plys E., Seward M., Allen E.M., Tatar R.G., Huberty J., Vranceanu A.M. (2025). Pilot Randomized Controlled Trial of the Feasibility of a Mobile App-Delivered Mindfulness-Based Intervention for Caregiver Stress. Gerontology.

[41] Risch A.K., Lechner-Meichsner F., Wilz G. (2025). Telephone-Based Acceptance and Commitment Therapy for Caregivers of Persons with Dementia: Results of a Randomized Controlled Trial. Clin. Gerontol..

[42] Shaw C.A., Williams K.N., Perkhounkova Y., Hein M., Coleman C.K. (2020). Effects of a Video-based Intervention on Caregiver Confidence for Managing Dementia Care Challenges: Findings from the FamTechCare Clinical Trial. Clin. Gerontol..

[43] Williams K.N., Perkhounkova Y., Shaw C.A., Hein M., Vidoni E.D., Coleman C.K. (2019). Supporting Family Caregivers with Technology for Dementia Home Care: A Randomized Controlled Trial. Innov. Aging.

[44] Stara V., Rampioni M., Moșoi A., Kristaly D., Moraru S.-A., Paciaroni L., Paolini S., Raccichini A., Felici E., Rossi L. (2022). A Technology-Based Intervention to Support Older Adults in Living Independently: Protocol for a Cross-National Feasibility Pilot. Int. J. Environ. Res. Public Health.

[45] Morgan M., Butow P., Maddern R., Shaw J. (2015). The Role of the Prostate Cancer Nurse Co-Ordinator: Nurses’ Perspectives of Barriers and Challenges. Int. J. Urol. Nurs..

[46] Green E., Quilliam C., Sheepway L., Hays C.A., Moore L., Rasiah R.L., Bailie J., Howard C., Hyde S., Inyang I. (2022). Identifying Features of Quality in Rural Placements for Health Students: Scoping Review. BMJ Open.

[47] Shaban M., Shaban M.M., Mohammed H.H., El-kest H.R.A. (2024). Barriers and Facilitators to Effective Pain Management in Elderly Arab Patients: A Nursing Perspective through a Qualitative Study. BMC Nurs..

[48] Badawy W., Shaban M. (2025). Exploring Geriatric Nurses’ Perspectives on the Adoption of AI in Elderly Care a Qualitative Study. Geriatr. Nurs..

[49] Badawy W., Zinhom H., Shaban M. (2025). Navigating Ethical Considerations in the Use of Artificial Intelligence for Patient Care: A Systematic Review. Int. Nurs. Rev..

[50] Seng J.J.B., Nyanavoli H., Decruz G.M., Kwan Y.H., Low L.L. (2025). Health Coaching and Its Impact in the Remote Management of Patients with Type 2 Diabetes Mellitus: Scoping Review of the Literature. J. Med. Internet Res..

[51] Jia E., Macon J., Doering M., Abraham J. (2025). Effectiveness of Digital Behavioral Activation Interventions for Depression and Anxiety: Systematic Review and Meta-Analysis. J. Med. Internet Res..

[52] Malik K., Ibrahim M., Bernstein A., Venkatesh R.K., Rai T., Chorpita B., Patel V. (2021). Behavioral Activation as an ‘Active Ingredient’ of Interventions Addressing Depression and Anxiety among Young People: A Systematic Review and Evidence Synthesis. BMC Psychol..

[53] Oberlin L.E., Jaywant A., Wolff A., Gunning F.M. (2022). Strategies to Promote Cognitive Health in Aging: Recent Evidence and Innovations. Curr. Psychiatry Rep..

[54] Badawy W.B.M., Mohamed A.H., Shaban M. (2024). Effectiveness of a Resilience-Building Nursing Intervention on Psychological Well-Being in Arab Community-Dwelling Older Adults. Geriatr. Nurs..

[55] McCurry S.M., Song Y., Martin J.L. (2015). Sleep in Caregivers. Curr. Opin. Psychiatry.

[56] Mukherjee U., Sehar U., Brownell M., Reddy P.H. (2024). Mechanisms, Consequences and Role of Interventions for Sleep Deprivation: Focus on Mild Cognitive Impairment and Alzheimer’s Disease in Elderly. Ageing Res. Rev..

[57] Ju E., Guo Y., Park J.I., Kim J., Qu A., Lee J.-A. (2025). Sleep Quality of Persons with Dementia and Family Caregivers in Korean Americans: Wearable Technology to Study the Dyadic Association. West. J. Nurs. Res..

[58] Mohamed S.A.A.K., Shaban M. (2024). Age and Expertise: The Effects of Ageism on Professional Recognition for Senior Nurses. Geriatr. Nurs..

[59] Christie H.L., Martin J.L., Connor J., Tange H.J., Verhey F.R.J., de Vugt M.E., Orrell M. (2019). EHealth Interventions to Support Caregivers of People with Dementia May Be Proven Effective, but Are They Implementation-Ready?. Internet Interv..

[60] Sani T.P., Cheung G., Peri K., Yates S., Kerse N., Whaanga H., Cullum S. (2025). Cultural Adaptations of the WHO ISupport for Dementia: A Scoping Review. Dementia.

[61] shaban M., Mohammed H.H., Amer F.G.M., Elsayed H.H., Ali S.I., Ibrahim A.M. (2024). Psychometric Evaluation of the Translated Arabic Version of the Geriatrics Health Behavior Questionnaire (GHBQ) for Geriatric Nurses: A Cross-Sectional Study. BMC Nurs..

[62] Wei J., Margetis G. (2025). Human-Centered Design, Operation and Evaluation of Mobile Communications.

[63] Jongebloed H., Anderson K., Winter N., Nguyen L., Huggins C.E., Savira F., Cooper P., Yuen E., Peeters A., Rasmussen B. (2024). The Digital Divide in Rural and Regional Communities: A Survey on the Use of Digital Health Technology and Implications for Supporting Technology Use. BMC Res. Notes.

[64] Dou J., Wang J., Gao X., Wang G., Bai Y., Liang Y., Yang K., Yang Y., Zhang L. (2025). Effectiveness of Telemedicine Interventions on Motor and Nonmotor Outcomes in Parkinson Disease: Systematic Review and Network Meta-Analysis. J. Med. Internet Res..

[65] Özden F. (2023). The Effect of Mobile Application-Based Rehabilitation in Patients with Parkinson’s Disease: A Systematic Review and Meta-Analysis. Clin. Neurol. Neurosurg..

[66] Hirczy S., Zabetian C., Lin Y.-H. (2024). The Current State of Wearable Device Use in Parkinson’s Disease: A Survey of Individuals with Parkinson’s. Front. Digit. Health.

[67] Shah S.P., Glenn G.L., Hummel E.M., Hamilton J.M., Martine R.R., Duda J.E., Wilkinson J.R. (2015). Caregiver Tele-Support Group for Parkinson’s Disease: A Pilot Study. Geriatr. Nurs..

[68] Alruwaili A.N., Alruwaili M.M., Ramadan O.M.E., Ali S.I., Shaban M. (2024). Nursing Strategies for Enhancing Calm in Older Arabs with Dementia: Integrating Snoezelen Methods, Aromatherapy, and Personal Items to Reduce Agitation. Geriatr. Nurs..

[69] Woolham J., Freddolino P., Gibson G., Daniels S. (2021). Telecare at a Crossroads? Finding Researchable Questions. J. Enabling Technol..

[70] Cheung K.S., Lau B.H., Wong P.W., Leung A.Y., Lou V.W.Q., Chan G.M., Schulz R. (2015). Multicomponent Intervention on Enhancing Dementia Caregiver Well-being and Reducing Behavioral Problems among Hong Kong Chinese: A Translational Study Based on REACH II. Int. J. Geriatr. Psychiatry.

[71] Elsayed Ramadan O.M., Alruwaili M.M., Alruwaili A.N., Elsharkawy N.B., Abdelaziz E.M., Zaky M.E., Shaban M.M., Shaban M. (2024). Nursing Practice of Routine Gastric Aspiration in Preterm Infants and Its Link to Necrotizing Enterocolitis: Is the Practice Still Clinically Relevant?. BMC Nurs..

[72] Nashwan A.J., Abou Hashish E.A., Mohamed A.S., Alrimawi I., Aqtam I., Al Obeisat S., Alhalaiqa F., Alzaatreh M., Al Hadidi M., AL-Fayyadh S. (2024). Exploring the National Nursing Research Priorities in the Eastern Mediterranean Region and Overcoming the Associated Challenges: An Expert Opinion. Cureus.

[73] Page M.J., McKenzie J.E., Bossuyt P.M., Boutron I., Hoffmann T.C., Mulrow C.D., Shamseer L., Tetzlaff J.M., Akl E.A., Brennan S.E. (2021). The PRISMA 2020 statement: An updated guideline for reporting systematic reviews. BMJ.

