# Supplementary material for: Home-Based Digital Healthcare Interventions for Dementia: A Systematic Review of Patient and Family Caregiver Outcomes
Source: Healthcare (Basel). 2026 Mar 27;14(7):854. doi: 10.3390/healthcare14070854 (PMC13073384; doi:10.3390/healthcare14070854)
Supplement: Supplementary file 1 [file healthcare-14-00854-s001.zip › healthcare-4163193-Supplementary Materials S1.pdf]

Supplementary Materials S1

Complete Electronic Search Strategy

This appendix provides the complete electronic search strategy used for all databases. The strategy combined controlled vocabulary (e.g., MeSH, Emtree) and free-text terms across four conceptual domains: (1) Dementia, (2) Digital/Technology-Based Interventions, (3) Home/Community Setting, and (4) Caregiver/Patient Outcomes.

1. PubMed (MEDLINE)

(("Dementia"[Mesh] OR "Alzheimer Disease"[Mesh] OR dementia[tiab] OR "Alzheimer*"[tiab] OR "major neurocognitive disorder"[tiab]) AND ("Telemedicine"[Mesh] OR "Remote Consultation"[Mesh] OR "Mobile Applications"[Mesh] OR "Digital Health"[tiab] OR eHealth[tiab] OR mHealth[tiab] OR telehealth[tiab] OR telemedicine[tiab] OR "remote monitoring"[tiab] OR "smart home"[tiab] OR wearable*[tiab] OR app*[tiab] OR robot*[tiab] OR "conversational agent"[tiab]) AND ("Home Care Services"[Mesh] OR "Community Health Services"[Mesh] OR "home-based"[tiab] OR "home care"[tiab] OR "community dwelling"[tiab]) AND ("Caregivers"[Mesh] OR caregiver*[tiab] OR "caregiver burden"[tiab] OR stress[tiab] OR sleep[tiab] OR "quality of life"[tiab] OR NPI[tiab] OR "Zarit"[tiab] OR PSQI[tiab]))

2. Embase (Elsevier)

('dementia'/exp OR 'alzheimer disease'/exp OR dementia:ti,ab OR alzheimer*:ti,ab OR 'major neurocognitive disorder':ti,ab) AND ('digital health'/exp OR 'telemedicine'/exp OR 'ehealth'/exp OR 'mhealth'/exp OR 'remote monitoring'/exp OR 'smart home'/exp OR wearable*:ti,ab OR app*:ti,ab OR robot*:ti,ab OR 'conversational agent':ti,ab) AND ('home care'/exp OR 'community dwelling'/exp OR 'home-based':ti,ab) AND ('caregiver'/exp OR caregiver*:ti,ab OR 'caregiver burden'/exp OR stress:ti,ab OR sleep:ti,ab OR 'quality of life'/exp)

3. CINAHL (EBSCOhost)

(MH "Dementia+") OR (MH "Alzheimer's Disease+") OR TI dementia OR AB dementia AND (MH "Telehealth+") OR (MH "Mobile Applications+") OR TI ("digital health" OR telehealth OR mhealth OR ehealth OR "remote monitoring" OR wearable OR app OR robot) AND (MH "Home Care Services+") OR TI ("home-based" OR "community dwelling") AND (MH "Caregivers+") OR TI ("caregiver burden" OR stress OR sleep OR "quality of life")

4. PsycINFO (APA)

(DE "Dementia" OR DE "Alzheimer's Disease" OR dementia OR Alzheimer*) AND (DE "Telemedicine" OR DE "Mobile Applications" OR DE "Health Information Technology" OR "digital intervention" OR telehealth OR mhealth OR ehealth OR "remote monitoring") AND ("home care" OR "home-based" OR "community dwelling") AND (DE "Caregivers" OR "caregiver burden" OR stress OR sleep OR "quality of life")

5. Scopus (Elsevier)

TITLE-ABS-KEY (dementia OR "Alzheimer disease" OR "major neurocognitive disorder") AND TITLE-ABS-KEY ("digital health" OR ehealth OR mhealth OR telehealth OR "remote

monitoring" OR "smart home" OR wearable OR app OR robot) AND TITLE-ABS-KEY ("home-based" OR "home care" OR "community dwelling") AND TITLE-ABS-KEY ("caregiver burden" OR stress OR sleep OR "quality of life")

6. Web of Science (Clarivate)
TS=(dementia OR "Alzheimer disease" OR "major neurocognitive disorder") AND TS=("digital health" OR ehealth OR mhealth OR telehealth OR "remote monitoring" OR "smart home" OR wearable OR app OR robot) AND TS=("home-based" OR "home care" OR "community dwelling") AND TS=("caregiver burden" OR stress OR sleep OR "quality of life")

Search limits: English language; human subjects; publication years January 2000 – October 2025. No initial date restrictions were applied during preliminary search development.
